# Recent Achievements and Perspectives in Nebulization Devices for Anterior Segment Disease Treatment

**DOI:** 10.3390/pharmaceutics18040404

**Published:** 2026-03-25

**Authors:** Hongru Liu, Qibin Deng, Jun Cao, Tao Wang, Junxi Chen, Ke Xiong

**Affiliations:** 1 School of Biomedical Sciences, Hunan University, Changsha 410082, China; liulie@seemore.com.cn (H.L.); caojun10@126.com (J.C.); wt15232232602@163.com (T.W.); 2 Greater Bay Area Institute for Innovation, Hunan University, Guangzhou 511300, China; 3 Shenzhen Shuimu Biopharmaceutical Co., Ltd., Shenzhen 518126, China; 4 Nanfang Hospital, Southern Medical University, Guangzhou 510515, China; 15170809654@163.com (Q.D.); 3220010020@i.smu.edu.cn (J.C.)

**Keywords:** nebulization devices, anterior segment, ophthalmic drug-delivery, ocular barriers, ocular bioavailability

## Abstract

Ocular diseases pose significant therapeutic challenges due to the eye’s intricate anatomy and efficient physiological clearance mechanisms, which result in the rapid elimination of topically administered drugs and an overall bioavailability of less than 5%. Anterior segment disorders—including keratitis, glaucoma, and dry eye syndrome—account for the majority of ophthalmic conditions and are primarily managed with pharmacological agents. However, due to extremely low drug bioavailability and poor patient compliance, their therapeutic outcomes often result in a decreased disease control rate or require early surgical interventions. Nebulized drug delivery, particularly employing advanced vibrating mesh technology, has emerged as a promising strategy to overcome these limitations. By converting liquid formulations into a uniform aerosol of micron-sized (1–10 μm) droplets, this approach achieves extensive and consistent coverage of the ocular surface, increases the absorption contact area, prolongs drug residence time, and ultimately enhances drug bioavailability. Preliminary clinical evidence indicates that nebulized therapies outperform traditional eye drops by achieving higher drug concentrations in the aqueous humor and demonstrating superior pharmacodynamic profiles and patient tolerability—particularly in conditions such as dry eye syndrome and glaucoma. This review presents a comprehensive overview of the mechanistic principles, technological advancements, and translational applications of nebulization-based ocular drug delivery systems. We place special emphasis on the integration of next-generation platforms that incorporate microelectromechanical systems (MEMS) and intelligent sensing technologies, enabling precision medicine approaches tailored to individual ocular pathophysiological characteristics. By bridging biomedical engineering and clinical ophthalmology, these innovations not only optimize existing therapeutic regimens but also pave the way for non-invasive delivery of complex biologics and gene therapies—potentially reshaping the landscape of anterior segment drug delivery.

## 1. Introduction

**Figure 1:**
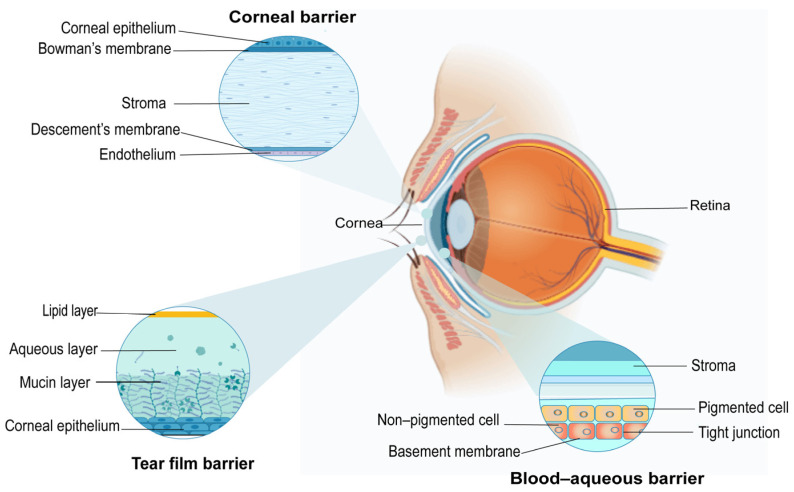
Barriers of ocular drug delivery.

The eye, often referred to as a “highly specialized organ”, plays a critical role in human perception and interaction with the external environment. Ocular diseases can severely impair vision and significantly diminish patients’ quality of life. According to global estimates, approximately 596 million individuals were affected by distance vision impairment in 2020, among whom 43 million were classified as blind [1]. Among anterior segment disorders, cataracts remain the primary cause of blindness worldwide. In contrast, other anterior segment disorders, such as dry eye disease and keratitis, are predominantly managed with pharmacological interventions. Anatomically, the eye is divided into the anterior and posterior segments. The anterior segment encompasses the conjunctiva, cornea, anterior chamber, iris, pupil, ciliary body, and lens. The unique architecture of each layer presents substantial challenges to ocular drug delivery. Anterior segment diseases, such as dry eye syndrome, keratitis, and iridocyclitis, are predominantly treated with topical medications (e.g., ophthalmic drops), and surgical interventions are indicated in certain severe cases, such as severe cataracts and glaucoma. However, the clinical efficacy of conventional topical ophthalmic formulations remains markedly limited due to suboptimal tissue penetration and rapid precorneal clearance (tear turnover, aqueous humor circulation, and drainage), resulting in extremely low ocular bioavailability (Figure 1).

It is estimated that less than 5% of topically administered drugs reach the aqueous humor. This pharmacokinetic inadequacy represents the most urgent clinical challenge [2]. This limitation necessitates frequent dosage regimens that can significantly increase patient burden and compromise compliance, particularly in elderly populations. More critically, the inability to sustain therapeutic drug levels in the anterior chamber and cornea often leads to suboptimal outcomes in severe conditions such as infectious keratitis or refractory glaucoma, where delayed or insufficient treatment can result in permanent vision loss. Therefore, there is an imperative need to develop delivery strategies that can overcome these barriers without relying solely on increasing drug concentration.

In response to these challenges, researchers have focused on developing advanced drug delivery strategies that surmount the anatomical and physiological barriers of ocular tissues. Among these strategies, novel delivery platforms such as nanoparticles, hydrogels, microneedles, and nebulization devices have been investigated to enhance drug permeation, retention, and targeted accumulation within the eye. Recent advances in nebulization devices have emerged as a transformative strategy. Nebulized eye drop formulations—by generating stable micron-scale (such as 1–10 μm) aerosolized particles—facilitate uniform distribution across the corneal and conjunctival surfaces, thereby increasing the effective contact area and improving drug absorption. The mild, non-invasive nature of nebulized administration minimizes drug loss associated with reflex blinking and lacrimation. Nebulization technology not only facilitates the repurposing and optimization of existing ophthalmic drugs for localized delivery but also reduces first-pass metabolism and systemic side effects.

In this narrative review, we primarily elucidate the anatomical and physiological structures most relevant to topical drug delivery in the anterior segment, conduct a comparative analysis of nebulization devices, and investigate the clinical applications of nebulization devices in anterior segment diseases. Given the limitations of existing treatment methods, we highlight the successful applications of nebulization devices in treating anterior segment diseases and summarize their advantages and challenges.

With the development of micro-electromechanical systems (MEMS) and intelligent sensors, the integration of these technologies into nebulization devices can achieve personalized and data-driven precision in ophthalmic treatment. Next-generation nebulization devices not only optimize the therapeutic performance of existing ophthalmic drugs but could also enable novel non-invasive delivery routes for new biological agents and gene therapies. Nebulization technology may become an emerging approach for treating eye diseases [2].

In our research, a structured literature search was conducted across the databases PubMed, Web of Science, and Google Scholar, covering studies published from 1990 to 2026. Search Strategy: Keywords used in various combinations included the following: (1) Device Technology—“ophthalmic nebulization,” “Vibrating mesh nebulizer,” “ultrasonic nebulizer,” “jet nebulizer,” and “aerosol generation”; (2) Ocular Administration—“Topical ocular delivery,” “ocular drug delivery,” “aerosol delivery to the eye,” “intraocular penetration, “ “thickness of tear film,” and “pre-corneal residence time”; (3) Clinical Application—“Enhanced ocular bioavailability,” “Dry eye syndrome,” “glaucoma mist therapy,” “infectious keratitis,” and “Vitamin B12 nebulization”.

The Inclusion Criteria included: (1) peer-reviewed clinical and preclinical studies investigating nebulized delivery specifically for ocular surfaces or the anterior segment; (2) studies comparing nebulization with traditional ophthalmic drops; (3) technical reports on nebulizer design relevant to ophthalmology; and (4) translational challenges in nebulized drug delivery to the eye. Preference was given to studies that provided empirical data on ocular bioavailability or quantitative comparisons between traditional eye drops and aerosolized formats. The Exclusion Criteria included: (a) studies focusing solely on pulmonary nebulization with no relevance to ocular delivery; (b) non-English-language publications; (c) white papers with no clinical data; and (d) papers where the nebulization device technology was not specified. A total of approximately 80 initially identified sources were screened, resulting in the final selection of references cited in this review, prioritizing the most recent high-quality clinical data.

## 2. Barriers to Ocular Drug Delivery and Nebulization Devices

**Figure 2:**
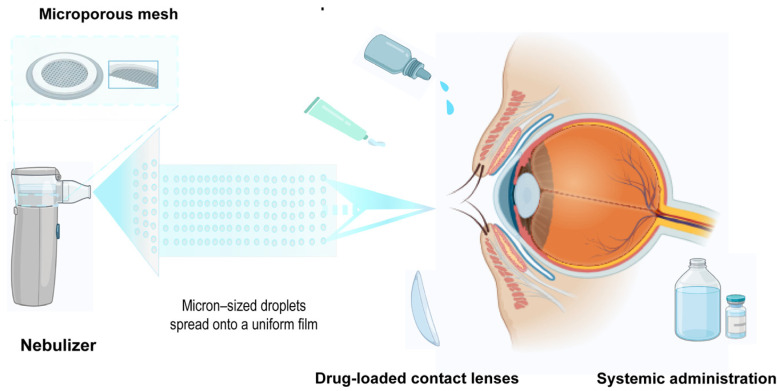
Drug delivery approaches in ophthalmology.

To understand why nebulization technology emerges as a transformative solution, it is first necessary to conduct an in-depth examination of the complexity of the eye as a drug delivery target and its intrinsic defense mechanisms. In this chapter, we provide a comprehensive overview of the anatomical and physiological barriers of the anterior segment. We subsequently elaborate on the core principles of nebulization technology and its theoretical underpinnings for addressing the challenges in ocular drug administration (Figure 2).

### 2.1. Anterior Segment

The anterior segment exhibits complex morphological organization and plays a pivotal role in maintaining the stability of the intraocular environment. For drugs to reach their target sites, they must surmount multiple barriers. These barriers can be generally classified into two types: anatomical barriers and physiological clearance mechanisms. These two categories function synergistically, leading to the “failure cascade” challenge in the administration of traditional eye drops.

#### 2.1.1. Anatomical Structure

The structures most pertinent to topical drug delivery in the anterior segment encompass the cornea, the conjunctiva, and the overlying tear film, each presenting distinct anatomical and physiological barriers that critically determine drug bioavailability.

The cornea, as the primary pathway for light to enter the eyeball, also serves as the principal route for drugs to reach the aqueous humor. It comprises a multi-layered structure, with its unique “sandwich” architecture being pivotal: the outer epithelium and inner endothelium are lipophilic, while the intermediate stroma—constituting 80–85% of total corneal thickness—is hydrophilic [3]. This lipophilic–hydrophilic–lipophilic biphasic property places stringent demands on drug permeability. Only amphiphilic drugs with both hydrophilic and lipophilic properties can permeate effectively, whereas purely hydrophilic or lipophilic drugs are respectively hindered by the epithelial layer or stromal layer [4].

The conjunctiva is a thin membrane lining the inner surface of the eyelid and the anterior sclera, with a surface area considerably larger than that of the cornea. As a mucosal tissue, the conjunctiva extends from the mucocutaneous junction at the eyelid margin to the corneal periphery, attaching to the sclera. It contains a dense network of blood and lymphatic vessels, resulting in most drugs absorbed via the conjunctiva entering the bloodstream rather than reaching intraocular lesion sites. This absorption process, termed “non-productive absorption”, not only markedly reduces drug bioavailability but can also induce systemic side effects [5].

The precorneal tear film is a liquid layer (2–6 µm thick) overlying the corneal and conjunctival surfaces, functioning as the first barrier encountered by drugs entering the eye. The tear film comprises three layers, from the outermost to the innermost: the lipid layer, aqueous layer, and mucin layer [6,7]. The tear film harbors proteins such as lysozyme and albumin, as well as various metabolic enzymes. These components can bind to drugs or degrade them, ultimately leading to a substantial reduction in drug concentration [8].

#### 2.1.2. Physiological Barriers and Clearance Mechanisms

Ocular dynamic physiological processes (tear turnover, blinking) constitute a key factor underlying the poor efficacy of topical eye drop therapy, which trigger a rapid “failure cascade” effect.

The normal volume of the conjunctival sac in healthy human eyes is only approximately 7 μL, whereas each commercially available ophthalmic drop typically has a volume of around 30 μL. This marked volumetric mismatch causes the majority of the instilled solution to efflux directly from the eye upon administration, resulting in significant waste [9]. High-volume formulations, those inducing a pronounced foreign body sensation, and irritant solutions immediately trigger a vigorous blink reflex and reflex tearing, which accelerate drug clearance and further compromise ocular bioavailability. Under normal conditions, the tear turnover rate is approximately 15% per minute, doubling post-instillation. Accelerated tear turnover further dilutes residual ocular formulation, leading to a significant reduction in drug concentration [10,11]. Residual formulation in the conjunctival sac rapidly drains into the nasal cavity and pharynx via the nasolacrimal duct system, entering systemic circulation. The excess volume dictates a high initial drainage rate, leading to the rapid elimination of over 95% of the dose via the nasolacrimal duct within 30 s. This results in corneal bioavailability of ophthalmic drop-administered drugs being less than 5% and may also induce unwanted systemic adverse effects [12]. By reducing the droplet size to the micron scale, aerosolized delivery provides a mass transfer at a rate that does not exceed the natural tear turnover (0.5–2.2 µL/min) [13,14]. This allows the medication to accumulate on the ocular surface as a “thin film” rather than a “bolus,” effectively bypassing the volumetric trigger for reflex blinking and drainage. Therefore, nebulization devices, by improving localized deposition and reducing excess dosing, may mitigate systemic absorption.

### 2.2. Principles of Ophthalmic Pharmacokinetics

Pharmacokinetics (PK) encompasses the absorption, distribution, metabolism, and excretion of drugs in the body. Key parameters of relevance include: the maximum drug concentration in target tissues (C max), time to reach this maximum concentration (T max), the area under the drug concentration–time curve (AUC), and the half-life (t1/2). Sampling from human intraocular tissues poses ethical and technical challenges; these parameters are typically obtained from animal models, notably rabbit models, with rabbits having relatively large eyeballs with a structure similar to that of the human eye [12]. Factors such as the degree of drug mixing with the corneal anterior tear film, ocular residence time, and drug concentration in the corneal anterior region determine ocular drug absorption efficiency.

In contrast to traditional ophthalmic drops, which are typically administered in supra-physiological volumes (normally 30 μL), far exceeding the conjunctival sac’s 7 μL volume, nebulized micro-droplets (1–10 µm) facilitate the maintenance of a saturated or near-saturated state (S_0_) on the thin tear film. According to the modified Fick’s Law (J = P⋅C⋅γ), where J is the saturation and flux, P is the drug permeability coefficient, C is the drug concentration, and γ is the activity coefficient, the high surface-area-to-volume ratio of the aerosol maximizes the effective concentration at the aqueous/lipid interface, promoting higher flux across the physiological barriers compared to the pulsed and diluted concentration typical of drops [15]. Micron-sized droplets generated by atomization spread into a uniform film across the entire corneal and conjunctival surfaces, significantly increasing the contact area between the drug and absorptive tissues and enhancing absorption efficiency. The dosage of nebulized administration is precisely controllable; its volume is usually much smaller than that of traditional ophthalmic drops. This mild administration method effectively avoids or alleviates blink reflexes and reflex tearing caused by a foreign body sensation. It also reduces the rate of decline of drug concentration in the tear film through continuous nebulization, facilitating prolonged effective retention of the drug on the ocular surface. Studies have shown that reducing ophthalmic drop volume may enhance drug bioavailability. Nebulization technology strategically leverages this principle, providing more drug molecules the opportunity to penetrate the corneal barrier through approaches of “small-volume, multiple administrations” and “film coverage”. A clinical study demonstrated that after vitamin B12 administration via nebulizer, the vitamin was detectable in the aqueous humor of 29% of subjects, while no detection was observed in those administered the same concentration of ophthalmic drops. This finding provides robust clinical evidence that nebulization technology significantly improves intraocular bioavailability [16].

### 2.3. Nebulization Devices

**Figure 3:**
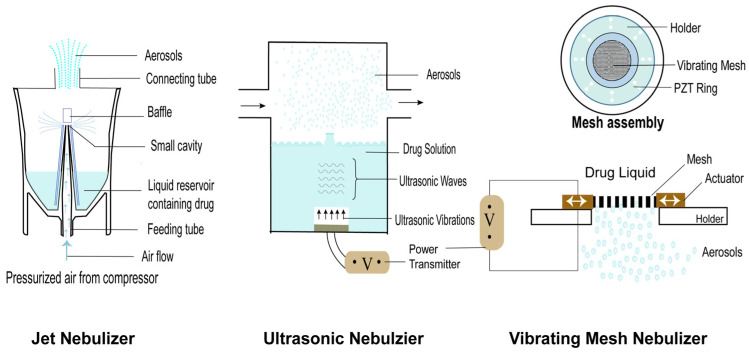
Mechanisms of nebulization devices.

According to aerosol generation principles, nebulizers are primarily classified into three types: jet nebulizers, ultrasonic nebulizers, and vibrating screen nebulizers. To meet the increasing and diverse needs of humans, nebulization devices have been continuously updated and iterated in line with advances over time (Figure 3).

#### 2.3.1. Jet Nebulizers

The jet nebulizer is the most conventional type of nebulizer, with its operating principle based on the Venturi and Bernoulli principles. When high-speed airflow generated by the compressor passes through the narrow nozzle, negative pressure is formed, and the medication solution in the reservoir cup is sucked out and sheared into large droplets. Large droplets impinge on the baffle; larger particles are intercepted and reflux. Small particles (1–5 μm) form aerosols and are emitted [17,18]. The key advantages of jet nebulizers are their status as a well-established technology and low cost. However, their limitations are notable. The equipment is bulky (reducing portability), requires an external compressor or air source, is user-unfriendly, and generates substantial noise during operation [18]. For ophthalmic applications, a key non-negligible limitation resides in their “evaporative cooling effect”. Continuous high-velocity gas flow under low ambient humidity promotes solvent evaporation (typically water), leading to a temperature reduction of the drug solution within the reservoir. During treatment, drug concentration rises incrementally, which compromises precise dosage control, especially in the late treatment stage [18].

#### 2.3.2. Ultrasonic Nebulizers

The ultrasonic nebulizer utilizes the converse piezoelectric effect to convert the alternating current into high-frequency (1–2.5 MHz) acoustic energy. This high-frequency acoustic energy acts directly on the medication solution, triggering the “acoustic energy fountain” phenomenon at the liquid surface and subsequently generating aerosols [18]. Compared with jet nebulizers, the key advantages of ultrasonic nebulizers are that they are quieter, are more portable, and have a higher nebulization rate. They also have non-negligible limitations. The medication solution is heated by the high-intensity acoustic energy they generate, causing heat-sensitive drugs such as proteins and peptides in biological preparations to denature and become inactive [17]. Suspended drug particles cannot be effectively atomized, which limits the application range of ultrasonic nebulizers.

#### 2.3.3. Vibrating Mesh Nebulizers

To meet the demands in drug administration, vibrating mesh technology has emerged and is currently the most advanced atomization technology. With the growing emergence of biological preparations such as proteins and peptides for ocular use, nebulization requirements include not only high efficiency but also the preservation of drug activity, which is of vital importance. Due to the heat generation problem, traditional ultrasonic nebulizers struggle to meet this demand. Against this backdrop, vibrating mesh nebulizer (VMN) technology emerged. Sustained, high-frequency membrane displacement forces liquid through thousands of lithographical micro-apertures, producing droplet ejection. Because energy is delivered through controlled solid–liquid displacement at the aperture rather than by bulk gas flow or high-intensity acoustic fields, volumetric heating is minimal and local temperature rise is limited. The core component of a VMN is a mesh, covered with thousands of precision microholes (diameter approximately 3–6 μm). The piezoelectric element drives the mesh or its surrounding transmission device to vibrate at high frequency, acting like a “micropump” to squeeze the medication solution out of the micropores, thereby forming an aerosol with uniform particle size, low velocity, and relatively mild characteristics [17]. The uniformity is derived from deterministic pore geometry, high pore density, synchronous vibration, aperture size and spacing.

VMNs not only incorporate the advantages of the previous two generations of technologies but also address their key limitations. In response to low nebulization efficiency and drug residue issues, VMNs have effectively addressed these problems. In addition, VMNs are typically battery-powered, compact, and user-friendly, facilitating easy transport by patients. They can precisely control aerosol particle size and generate uniform, stable aerosols [17]. Most importantly, VMNs operate with low energy consumption and generate almost no heat, making them particularly suitable for delivering heat-sensitive preparations such as biological macromolecules, suspensions, and liposomes [19]. These advantages not only enable VMNs to achieve incremental technical improvements but also expand new application approaches for the local ocular delivery of new biological drugs. To facilitate a clearer understanding of current device performance, Table 1. provides a comprehensive comparison of various nebulization technologies tailored for ophthalmic use.

## 3. The Application of Nebulization Devices in Anterior Segment Diseases

The preceding sections have discussed the theoretical advantages of nebulization technology over traditional eye drops. This chapter will examine in depth the existing preclinical and clinical research evidence, and summarize and discuss the practical application effects and potential of nebulized drug administration in the treatment of specific anterior segment diseases. We will compare nebulized administration with eye drops in terms of bioavailability, clinical efficacy, and patient experience. Preliminary clinical data regarding the ocular nebulized drug administration is presented in Table 2.

### 3.1. The Nebulization Device Enhances the Bioavailability of Drugs

The concentration of drugs in the eye is a direct factor determining therapeutic efficacy. Whether nebulization technology can translate its theoretical advantages into practice and truly enhance drug bioavailability is central to its clinical value. An innovative study conducted on cataract surgery patients has provided the most compelling clinical evidence to date for the advantages of nebulized drug administration [16]. In this study, patients were divided into three groups: one received vitamin B12 delivered via an ultrasonic nebulizer, another received vitamin B12 eye drops of the same concentration, and the third was a control group without any treatment. Twelve hours after administration, aqueous humor samples were collected and analyzed. The results showed that in the nebulization group, among 14 patients, 4 (29%) had a vitamin B12 concentration of 10^−7^ mol/L detected in their aqueous humor. In both the eye drop group and the control group, no vitamin B12 was detected in the aqueous humor. The superior results observed in the nebulized group are attributed to the uniform distribution of the mist across the ocular surface and the mitigation of reflex-mediated clearance, which allows for deeper and more persistent intraocular penetration even 12 h post-administration [16]. This experimental result suggests that for molecules such as vitamin B12, nebulized delivery can achieve intraocular penetration that is difficult for eye drops to attain, enabling the drug to reach the aqueous humor and exert its effect. This essentially confirms the core hypothesis that nebulization technology enhances intraocular bioavailability by comprehensively covering the ocular surface and prolonging drug retention time in the eye.

Another treatment experiment in glaucoma patients primarily compared the therapeutic effects of nebulized dorzolamide/timolol fixed combination (DTFC) with traditional eye drops [22]. The study found that in terms of intraocular pressure (IOP) reduction, significant decreases were observed at both 15 and 60 min post-administration. Notably, nebulized administration not only reduces IOP but also demonstrates significant advantages in improving fundus hemodynamics: it can more persistently increase blood flow velocity in the ophthalmic artery and central retinal artery, and significantly reduce the resistive index of the central retinal artery at 60 min. All patients receiving traditional eye drops reported ocular irritation, whereas only one patient in the nebulization group reported mild general discomfort. This indicates that nebulization technology enhances medication comfort [19]. This study reveals a more profound phenomenon: nebulized drug delivery may exert pharmacodynamic effects of distinct natures. It is not merely a “more efficient eye drop”. Its characteristics—low concentration, long duration, and large-area mild stimulation—stand in sharp contrast to the high-concentration, short-duration “shock” administration of traditional eye drops. This dual advantage of “efficacy combined with comfort” is significant for patients with chronic diseases like glaucoma requiring lifelong medication, ensuring good treatment compliance during long-term administration.

### 3.2. Application of Nebulization Device in Dry Eye Syndrome (DES)

Dry eye syndrome is a multifactorial disorder defined by the disruption of tear film homeostasis and accompanied by ocular discomfort. Its etiologies include reduced aqueous tear secretion from the lacrimal glands, accelerated evaporation of the aqueous phase of the tear film, and tear quality dysfunction [23]. In traditional treatment, artificial tears and anti-inflammatory agents are the most commonly employed methods, often with complex regimens [24]. While nebulization therapy possesses a unique advantage in “humidifying synergy” in the field of dry eye treatment. Nebulization therapy’s humidifying synergy not only stabilizes the tear film but also reduces evaporative loss, thereby lowering tear osmolarity and prolonging tear break-up time. These effects improve ocular surface homeostasis, alleviate discomfort, and enhance drug distribution and retention, offering more consistent therapeutic outcomes in dry eye management. In a clinical study of dry eye patients administered the neurotrophin vitamin B12 (VB12) and oxytocin (OXT), which has anti-inflammatory potential, via nebulization [25], both groups showed significant improvements in symptoms assessed using the Ocular Surface Disease Index (OSDI), as well as improved tear break-up time (BUT) and corneal fluorescein staining (CFS) compared with baseline, with prolongation of BUT (2.5–4.2 s), a reduction in OSDI scores (15–20 points), and tear osmolarity reduction (12–18 mOsm/L) [25]. The study authors note that nebulization therapy has several advantages over conventional eye drops: Continuous delivery of low-dose aerosolized medication (1 mL/min) to the site of action yields more consistent outcomes; due to its non-invasiveness and painlessness, patients with DES exhibit high compliance. It can simultaneously exert humidifying, anti-inflammatory, and neurotrophic effects, thereby targeting multiple etiologies of dry eye, and has potential for combination with other existing medications. It provides a potential approach for home-based treatment in dry eye patients [25].

### 3.3. Application of Nebulization Device in Keratitis

To date, no clinical trials have specifically investigated the use of nebulized antibiotics or antifungals in the treatment of keratitis. However, the current treatment approach poses significant challenges, and nebulization technology holds substantial theoretical advantages. Taken together, nebulization therapy exhibits considerable potential in the treatment of keratitis.

#### 3.3.1. Theoretical Basis for Nebulized Antimicrobial Agents

The standard treatment for severe bacterial keratitis is highly rigorous, typically requiring hospitalization and 24 h hourly administration of topical ophthalmic drops (e.g., vancomycin, tobramycin). These drops are highly concentrated, irritating, and often extemporaneously compounded in hospital pharmacies [26]. Such high-frequency, high-concentration regimens not only cause significant patient discomfort but also pose substantial challenges to treatment adherence and carry a potential risk of ocular surface toxicity. For fungal keratitis, due to poor drug permeability and low bioavailability, the treatment course is protracted and therapeutic efficacy is suboptimal [27]. The overall treatment success rate with topical agents was 63.12% (141/223), and mean healing time was 41.5 ± 22.2 days. Nebulization technology has the potential to substantially improve keratitis treatment. By forming a uniform drug film across the entire corneal surface, it may: (1) ensure complete, gap-free drug coverage of infectious foci; (2) enhance delivery efficiency, enabling lower, safer drug concentrations to achieve equivalent or superior efficacy; and (3) significantly reduce dosing frequency, alleviate patient burden, and improve treatment adherence [28]. Existing antibiotics (e.g., vancomycin, ciprofloxacin) and antifungal agents (e.g., amphotericin B, voriconazole) are potential candidates for nebulized formulations [26]. For ophthalmic agents to be suitable for nebulization, several physicochemical properties must be optimized. Nebulized formulations require adequate aqueous solubility to ensure stable droplet formation and prevent precipitation or nozzle clogging. Viscosity and surface tension must be controlled within ranges that permit efficient extrusion through micro-apertures, as excessive viscosity or high interfacial tension reduces aerosol output and alters droplet size distribution. Chemical stability is essential, since nebulization subjects formulations to mechanical vibration and potential shear stress. Particle size and homogeneity are critical: suspensions or poorly soluble drugs must be micronized and uniformly dispersed to avoid aggregation and ensure consistent aerosolization. In addition, isotonicity and pH buffering are required to maintain ocular tolerability, while excipients should be carefully selected to avoid ocular irritation or systemic absorption. To date, there remains significant scope for exploration in keratitis treatment. The development of novel drug delivery approaches holds promise for improving the challenging landscape of keratitis, which is characterized by refractoriness and suboptimal therapeutic outcomes [16].

#### 3.3.2. Integrating Nebulized Therapy with Novel Agents for Refractory Keratitis

In recent years, researchers have investigated the therapeutic efficacy of certain non-pharmacological therapies in refractory keratitis, among which photodynamic antimicrobial therapy (PDAT) and corneal cross-linking have shown unique advantages [28,29]. A prerequisite for the efficacy of these therapies is the uniform application of photosensitizers (e.g., rose bengal) to the corneal surface. As an ideal modality for uniform drug delivery, nebulization technology can also provide an optimal delivery platform to advance these emerging therapies.

### 3.4. Application of Nebulization Device in Glaucoma

The study of DTFC (dorzolamide–timolol fixed combination) nebulization mentioned above [22] has confirmed that nebulized administration can effectively reduce intraocular pressure, enhance ocular blood flow, and provide patients with a more comfortable medication experience. This is helpful for glaucoma patients requiring long-term IOP control and a comfortable medication experience to improve compliance. Caution is warranted in the selection of medications for glaucoma. A previous study showed that leakage of aerosolized anticholinergic agents (e.g., ipratropium bromide) into the eye when the mask is not tightly sealed can induce mydriasis and acute angle-closure glaucoma in predisposed individuals [30]. This underscores the need for ongoing attention to drug safety and careful medication selection to prevent adverse outcomes.

**Table 2:** Summary of preliminary clinical evidence from human studies regarding nebulized drug administration in ophthalmology.

Ocular Disorders	Nebulized Drug Categories	Types of Nebulizers Used	Key Comparison Results with Ophthalmic Drops
Bioavailability	VitaminB12 (Cyanocobalamin)	Ultrasonic nebulizer	A total of 29% of patients in the nebulized group had drug detected in the aqueous humor, versus 0% in the ophthalmic drops group [16].
Glaucoma	DTFC (dorzolamide–timolol fixed combination)	Vibrating mesh nebulizer	Equivalent efficacy in reducing IOP; the nebulized group exhibited significantly improved ocular blood flow with no ocular irritation [22].
Dry eye syndrome	VitaminB12/oxytocin	Ultrasonic Nebulizer	Significantly improves dry eye symptoms and signs [25].

## 4. Technological Advancements and Future Prospects

Ophthalmic nebulization technology is in the stage of technological integration and expanded applications. Currently, the evidence base is constrained by a lack of large-scale, multicenter randomized controlled trials. Future research must prioritize high-quality clinical data for vision-threatening diseases like infectious keratitis and refractory glaucoma to transform the current hypothesis-driven advantages into mainstream clinical standards. From innovations in fundamental nebulization mechanisms to the development of intelligent treatment platforms and the integration of microfabrication technology, these advancements are driving revolutionary progress in nebulization devices. Ophthalmic nebulization drug delivery is no longer in the conceptual stage but is advancing rapidly toward mainstream clinical and personalized treatment modalities. This section will discuss cutting-edge technologies and their potential roles in future ophthalmic treatment and diagnosis. The comparative advantages and operational trade-offs between nebulized delivery and alternative advanced systems are detailed in Table 3.

### 4.1. Emergence of Intelligent Nebulizers and Personalized Ophthalmic Care

The development trend of modern medicine is gradually moving toward the integration of digital technologies and medical devices, and nebulizers align with this trend. The next generation of smart nebulizers is being rapidly developed, advancing drug delivery from a passive process to a personalized treatment modality with precise control and traceability.

These next-generation smart nebulizers can achieve remote monitoring and data management via built-in Bluetooth modules, with data synchronization to mobile applications or cloud platforms [15]. Pressure sensors and other components are incorporated into the devices, enabling determination of whether they are correctly activated and employed in ophthalmic reagent administration [31]. They can also automatically and accurately record information such as time, date, treatment duration, device status, and battery level for each treatment [31], and provide real-time guidance for correct device operation via vibration (tactile feedback) or LED lights (visual cues), which may improve dosing reproducibility and reduce user error in anterior segment therapy.

Poor patient adherence is a key cause of treatment failure in chronic diseases. Intelligent nebulizers provide objective and non-tamperable medication records, enabling physicians to accurately ascertain patients’ actual medication status, conduct effective interventions, and provide education to improve therapeutic efficacy [32]. They can adjust the nebulization rate and set medication reminders via applications based on patients’ specific clinical conditions and treatment responses to achieve personalized treatment [33]. In clinical trials, intelligent nebulizers can eliminate bias stemming from reliance on patients’ subjective diaries, objectively provide real-world patient medication data, and significantly enhance the accuracy and reliability of trial outcomes.

Intelligent nebulizers have promising development potential and are expected to become valuable digital biomarker tools. Unlike conventional drug delivery devices, they are no longer limited to recording medication behaviors but can further convert such behavioral data into clinically relevant indicators. Taking dry eye patients as an example, when the usage frequency of intelligent nebulizers increases significantly over a specific period, this data serves as a precise signal—as an objective digital biomarker—clearly indicating that the disease may be in an acute exacerbation phase. Based on this, the system can automatically send alerts to clinicians. In this way, physicians can conduct timely early intervention even before patients’ subjective symptoms deteriorate significantly or prior to scheduled appointments, thereby markedly enhancing the timeliness and efficacy of treatment.

### 4.2. Application of Microelectromechanical System (MEMS) in Nebulizers

Microelectromechanical system (MEMS) refer to electromechanical devices at the micrometer scale. This technology is profoundly transforming ophthalmic medical devices: MEMS technology is used to manufacture the mesh—the core component of vibrating mesh nebulizers (VMNs). High-performance VMNs are significantly more expensive than traditional jet nebulizers due to the high manufacturing cost of their core component (the mesh) [34]. Compared with traditionally assembled metal meshes, MEMS VMNs thus couple finer pore-engineering with repeatable actuator–mesh mechanics to deliver tighter aerosol specifications, improved portability (thinner, lighter membranes), and a clearer path to unit cost reduction at volume through wafer-scale processes and reduced rework in final assembly. The application of MEMS technology is expected to reduce the manufacturing cost of VMNs, enabling the most advanced nebulization technology to transcend high-end market limitations and truly benefit all patients. Smart contact lenses incorporating MEMS pressure sensors have been developed, enabling 24 h continuous monitoring of intraocular pressure in glaucoma patients. Unlike methods relying on corneal compression (e.g., indentation, flattening, or rebound techniques), this technology measures intraocular pressure by sensing corneal curvature changes via sensors [35]. Thus, we can logically propose a new glaucoma treatment strategy: Patients wear MEMS-enabled monitoring contact lenses; when intraocular pressure reaches the therapeutic threshold, data is transmitted to the cloud via a Bluetooth-enabled device, prompting patients to receive nebulizer administration instructions. Post-administration, medication records are automatically uploaded. This forms a complete closed loop from detection to treatment and feedback, thereby achieving the goal of personalized treatment.

### 4.3. Application of Nebulization Devices in Advanced Treatment Protocols

To date, in the field of ocular disease treatment, numerous advanced therapies—including antibodies, peptides, nucleic acid drugs, and ocular gene therapies—have been employed. In the absence of effective non-invasive delivery approaches, intraocular injections, as a relatively invasive delivery route, remain necessary. The emergence of VMN technology undoubtedly offers a promising route for topical ocular surface delivery of these advanced therapies. Notably, in respiratory medicine, Pulmozyme^®^ (a formulation containing recombinant human deoxyribonuclease I (DNase)) has been successfully used in treating cystic fibrosis via nebulization. This success provides robust evidence for the feasibility and efficacy of aerosol-based delivery of biomacromolecules [36].

### 4.4. The Challenge of Clinical Translation

Although ophthalmic nebulization technology has broad prospects, challenges remain in its translation from laboratory to clinical practice. First, there are regulatory barriers. Nebulized drug delivery combines drugs and devices, and its approval process is considerably more complex than that for a single drug or device. Second, there is insufficient clinical trial evidence. To date, there is a paucity of high-quality clinical data. Large-scale randomized controlled trials (RCTs) are urgently needed to clarify the long-term efficacy and safety of nebulized ophthalmic formulations compared with gold standard ophthalmic drops across different diseases. Currently, nebulization is primarily utilized in pulmonary diseases [37]. Third, this technology has uncertain long-term safety. Although the nebulization device itself is safe, drugs may undergo systemic absorption via nasolacrimal drainage, necessitating comprehensive evaluation of ocular toxicity, systemic exposure, and age-specific risks. Current clinical reports focus primarily on acute or short-term effects. There is a complete lack of long-term data (beyond a few months) to assess chronic ocular surface changes or cumulative systemic absorption. The long-term impact of constant aerosol exposure on the corneal endothelium and conjunctival goblet cell density remains entirely unstudied. Thus, comprehensive long-term safety evaluations of nebulized dosing regimens are necessary, with attention paid to ocular adverse effects and monitoring of potential systemic reactions. Fourth, the technology has low cost-effectiveness and accessibility. Advanced intelligent nebulizers have a higher initial purchase cost than traditional ophthalmic drops. A key factor in patient acceptance is whether nebulization can offset the economic costs of the devices by reducing drug waste, improving efficacy, decreasing complications, or lowering long-term healthcare utilization [24].

Currently, nebulized drug administration is primarily used in respiratory diseases [37], with a lack of standardized administration protocols for ophthalmic diseases. Unlike pulmonary medicine, there are no standardized ophthalmic administration protocols. The lack of consensus on mesh size, exposure time, and particle dose across different studies makes it impossible to perform a meaningful meta-analysis at this time. Industry associations and regulatory agencies should take the lead in establishing standardized testing methods and evaluation criteria for assessing the performance of ophthalmic nebulizers. While early-stage pilot studies indicate potential benefits in localized drug deposition and patient comfort, these studies were often non-blinded or involved heterogeneous patient groups. Therefore, these results should be viewed as preliminary indicators of technical feasibility rather than definitive proof of therapeutic superiority. Ophthalmic nebulization currently lacks the high-level evidence required to challenge the established topical drops.

Based on the above analysis, to advance the treatment of ophthalmic diseases, the following issues need to be addressed: Commercial ophthalmic preparations cannot be directly nebulized, necessitating the development of drug formulations and dosage forms optimized for nebulized delivery. Dedicated formulations optimized for aerosolization considering physicochemical stability, droplet size distribution, and ocular retention are required.

Priority should be given to diseases that severely affect vision, exhibit suboptimal therapeutic outcomes, and require long-term medication—such as glaucoma, infectious keratitis, and severe dry eye syndrome—and to conducting high-quality clinical trials to obtain key efficacy and safety data.

To explore the integration of personalized and digital therapies, efforts should be made to actively facilitate the combination of intelligent nebulizers with digital health platforms, telemedicine, and artificial intelligence, and to develop comprehensive digital therapeutic solutions capable of providing personalized feedback, predicting disease progression, and optimizing treatment plans.

**Table 3:** Comparative analysis of nebulization versus established advanced delivery systems.

Delivery System	Key Advantages	Major Limitations	Key Results of Comparison with Nebulizer
Nanoparticles	Enhanced penetration; targeted delivery [38,39]	Complex formulation; potential cytotoxicity [39]	Nanoparticles improve depth of penetration [40]; nebulization improves uniformity and comfort [25]
Hydrogels	Extended residence time; highest bioavailability [41]	Minimally invasive; intracameral injection [41]	Gels provide longer duration [41]; nebulization provides a “mild, non-invasive” sensation with no blurring [25]
Drug-eluting contact lenses	Non-invasive; constant rate of release [42]	Material degradation; discomfort; risk of keratitis [42]	Contact lenses are superior for long-term dosing; nebulization is superior for patient-led, non-invasive acute dosing [25]

## 5. Conclusions

Although ophthalmic drops have long been the mainstay for treating anterior segment diseases, their bioavailability is often less than 5%, and patient adherence is generally poor, thereby failing to meet clinical expectations for efficacy and safety. Due to the combined effect of ocular anatomical and physiological barriers (e.g., tear dilution, blink-mediated clearance, and corneal barrier), most topically administered drugs are eliminated before exerting their effects, limiting their efficacy. Improving drug bioavailability has become a core challenge in advancing innovations in ophthalmic treatment strategies [39]. In this context, ophthalmic nebulization technology typified by the vibrating mesh nebulizer has emerged as a highly integrated solution to overcome existing limitations. By converting drug solutions into micron-scale, low-irritant aerosols, aerosol-based delivery not only redefines the interaction between the drug and the ocular surface but also significantly increases the effective contact area between the cornea and conjunctiva [43]. Compared with the protective clearance mechanism triggered by larger droplets in traditional ophthalmic drops, nebulization technology can achieve uniform drug deposition on the ocular surface via a gentler, quantifiable approach, resulting in significantly prolonged residence time and enhanced permeability.

Preliminary clinical evidence supports that the therapeutic efficacy of nebulized drugs is superior to that of ophthalmic drops; previous studies have shown that aerosol-based delivery not only exhibits a superior pharmacokinetic profile in terms of drug penetration but also significantly enhances patient comfort and adherence while maintaining equivalent or superior efficacy. These findings indicate that nebulization technology is more than merely an incremental optimization; rather, it is a novel treatment paradigm with potentially novel pharmacodynamic benefits [22].

Moving forward, the key driver in this field will stem from cross-technology convergence. The introduction of intelligent nebulization systems is integrating digital capabilities into traditional treatment methods. By integrating sensor networks, wireless connectivity, and real-time data analysis, nebulizer devices are evolving functionally from solely a drug delivery platform to a personalized healthcare system incorporating treatment, monitoring, interaction, and adaptive feedback [44]. Such systemic changes are expected not only to substantially enhance patient adherence and efficacy assessment but also to lay the foundation for developing next-generation data-driven, precise ophthalmic diagnosis and treatment systems.

## Figures and Tables

**Table 1:** Comparative analysis of nebulization technologies for ophthalmic applications.

Device Category	Jet Nebulizer	Ultrasonic Nebulizer	Vibrating Mesh Nebulizer
Mechanism	Bernoulli/Venturi effect [18]	Piezoelectric crystals generate an acoustic energy fountain through high-frequency vibration [19]	Piezoelectric elements drive the microporous mesh to extrude the medication solution [19]
Particle size	1–5 µm, non-uniform particle size distribution [17]	5–10 µm [17]	Precisely controllable; typically 3–6 µm; monodispersity [17]
Nebulization rate	Moderate	High	High
Topical administration time	10–15 min [19]	2–5 min [17]	2–5 min [13]
Heat generation	Cooling effect, resulting in solvent evaporation and increased medication solution concentration [17]	Significant heat generation, up to over 40 °C [17]	Virtually no heat generation [17]
Suitability for biological agents	Not suitable, shear force may affect activity	Not suitable, heat-induced protein denaturation [19]	Ideal; low energy consumption; no thermal damage [19]
Suitability for suspension	Suitable	Not suitable, cannot be effectively nebulized	Suitable
Portability and size	Poor; requires an external compressor; bulky in size	Good, but controller remains bulky	Excellent; compact; lightweight; battery-powered
Ophthalmic application advantages	Low cost	Quiet	High efficiency; precision; low residue; wide range of applicable drugs; portable
Ophthalmic application disadvantages	High noise; imprecise dosage; poor portability [20]	Heat generation limits drug selection; relatively large particles	Relatively high cost; mesh may be clogged by high-viscosity medication solutions [21]

## Data Availability

No new data were created or analyzed in this study. Data sharing is not applicable to this article as it is a review of the existing literature.

## References

[1] Burton M.J., Ramke J., Marques A.P., A Bourne R.R., Congdon N., Jones I., Tong B.A.M.A., Arunga S., Bachani D., Bascaran C. (2021). The *Lancet Global Health* Commission on Global Eye Health: Vision beyond 2020. Lancet Glob. Health.

[2] Del Amo Eva M. (2022). Topical ophthalmic administration: Can a drug instilled onto the ocular surface exert an effect at the back of the eye?. Front. Drug Deliv..

[3] Meek K.M., Knupp C. (2015). Corneal structure and transparency. Prog. Retin. Eye Res..

[4] Liu L.-C., Chen Y.-H., Lu D.-W. (2023). Overview of Recent Advances in Nano-Based Ocular Drug Delivery. Int. J. Mol. Sci..

[5] Geroski D.H., Edelhauser H.F. (2001). Transscleral drug delivery for posterior segment disease. Adv. Drug Deliv. Rev..

[6] Khanna R.K., Catanese S., Emond P., Corcia P., Blasco H., Pisella P.-J. (2022). Metabolomics and lipidomics approaches in human tears: A systematic review. Surv. Ophthalmol..

[7] Pflugfelder S.C., Stern M.E. (2020). Biological functions of tear film. Exp. Eye Res..

[8] Chang A.Y., Purt B. (2021). Biochemistry, tear film. StatPearls.

[9] Lanier O.L., Christopher K.G., Macoon R.M., Yu Y., Sekar P., Chauhan A. (2020). Commercialization challenges for drug eluting contact lenses. Expert Opin. Drug Deliv..

[10] Mofidfar M., Abdi B., Ahadian S., Mostafavi E., Desai T.A., Abbasi F., Sun Y., Manche E.E., Ta C.N., Flowers C.W. (2021). Drug delivery to the anterior segment of the eye: A review of current and future treatment strategies. Int. J. Pharm..

[11] Lee V.H.-L., Robinson J.R. (1979). Mechanistic and quantitative evaluation of precorneal pilocarpine disposition in albino rabbits. J. Pharm. Sci..

[12] Patel A., Cholkar K., Agrahari V., Mitra A.K. (2013). Ocular drug delivery systems: An overview. World J. Pharmacol..

[13] Knoch M. (2024). New Generation Nebulizers. J. Aerosol Med. Pulm. Drug Deliv..

[14] Sadeghi A., Subrizi A., del Amo E.M., Urtti A. (2024). Mathematical Models of Ocular Drug Delivery. Investig. Ophthalmol. Vis. Sci..

[15] Sripetch S., Loftsson T. (2021). Topical drug delivery to the posterior segment of the eye: Thermodynamic considerations. Int. J. Pharm..

[16] Kahn M. (2005). Bioavailability of vitamin B12 using a small-volume nebulizer ophthalmic drug delivery system. Clin. Exp. Ophthalmol..

[17] Ari A. (2014). Jet, Ultrasonic, and Mesh Nebulizers: An Evaluation of Nebulizers for Better Clinical Outcomes. Eurasian J. Pulmonol..

[18] Rau J.L. (2002). Design principles of liquid nebulization devices currently in use. Discuss. Respir. Care.

[19] Tservistas M., Fuchs C. Principles of Nebulizer Technology. https://www.inhalationmag.com/article/principles-nebulizer-technology/.

[20] Ashraf S., McPeck M., Cuccia A.D., Smaldone G.C. (2020). Comparison of Vibrating Mesh, Jet, and Breath-Enhanced Nebulizers During Mechanical Ventilation. Respir. Care.

[21] Ehrmann S. (2018). Vibrating Mesh Nebulisers—Can Greater Drug Delivery to the Airways and Lungs Improve Respiratory Outcomes?. Eur. Respir. Pulm. Dis..

[22] Janulevičienė I., Šiaudvytytė L., Baršauskaitė R., Dilienė V. (2013). The effect of nebulized dorzolamide/timolol fixed combination mist versus drops on retrobulbar blood flow and intraocular pressure in glaucoma patients. Medicina.

[23] Zemanová M. (2021). Dry eye disease. A review. Czech Slovak Ophtalmol..

[24] Messmer E.M. (2015). The pathophysiology, diagnosis, and treatment of dry eye disease. Dtsch. Arztebl. Int..

[25] Yang J., Liu Y., Xu Y., Li X., Fu J., Jiang X., Chou Y., Ma J., Hao R., Zhang R. (2019). A new approach of ocular nebulization with vitamin B12 versus oxytocin for the treatment of dry eye disease: An in vivo confocal microscopy study. Drug Des. Dev. Ther..

[26] Austin A., Lietman T., Rose-Nussbaumer J. (2017). Update on the Management of Infectious Keratitis. Ophthalmology.

[27] Sharma N., Bagga B., Singhal D., Nagpal R., Kate A., Saluja G., Maharana P.K. (2022). Fungal keratitis: A review of clinical presentations, treatment strategies and outcomes. Ocul. Surf..

[28] Su G., Wei Z., Wang L., Shen J., Liang Q. (2020). Evaluation of Toluidine Blue-Mediated Photodynamic Therapy for Experimental Bacterial Keratitis in Rabbits. Transl. Vis. Sci. Technol..

[29] Cherfan D., Verter E.E., Melki S., Gisel T.E., Doyle F.J., Scarcelli G., Yun S.H., Redmond R.W., Kochevar I.E. (2013). Collagen Cross-Linking Using Rose Bengal and Green Light to Increase Corneal Stiffness. Investig. Ophthalmol. Vis. Sci..

[30] Kalra L., Bone M.F. (1988). The Effect of Nebulized Bronchodilator Therapy on Intraocular Pressures in Patients with Glaucoma. Chest.

[31] Apstar Kolibri—The Digital Mesh Nebulizer System for Inhaled Drug Delivery. https://aptar.com/products/pharmaceutical/kolibri-nebulizer-soft-mist-inhaler/.

[32] Cuevas Brun E., Hsu Y. (2022). Advancements in Monitoring Adherence–A Smart Mesh Nebuliser. ONdrugDelivery.

[33] Nebulizers J. Smart Mesh Nebulizer by Briutcare. https://justnebulizers.com/products/smart-mesh-nebulizer-by-briutcare.

[34] Moon S.-H., Chang K.H., Park H.M., Park B.J., Yoo S.K., Nam K.C. (2021). Effects of Driving Frequency and Voltage on the Performances of Vibrating Mesh Nebulizers. Appl. Sci..

[35] Sanchez I., Martin R. (2019). Advances in diagnostic applications for monitoring intraocular pressure in Glaucoma: A review. J. Optom..

[36] Laube B.L. (2015). Aerosolized Medications for Gene and Peptide Therapy. Respir. Care.

[37] Boe J., Dennis J., O’driscoll B., Force M.O.T., Bauer T., Carone M., Dautzenberg B., Diot P., Heslop K. (2001). European Respiratory Society Guidelines on the use of nebulizers: Guidelines prepared by a European Respiratory Society Task Force on the use of nebulizers. Eur. Respir. J..

[38] Li J., Tian S., Tao Q., Zhao Y., Gui R., Yang F., Zang L., Chen Y., Ping Q., Hou D. (2018). Montmorillonite/chitosan nanoparticles as a novel controlled-release topical ophthalmic delivery system for the treatment of glaucoma. Int. J. Nanomed..

[39] Luo L.J., Nguyen D.D., Lai J.Y. (2020). Dually functional hollow ceria nanoparticle platform for intraocular drug delivery: A push beyond the limits of static and dynamic ocular barriers toward glaucoma therapy. Biomaterials.

[40] Hu J., Li H., Zhao Y., Ke Y., Rupenthal I.D., Liu H., Ye J., Han X., Yang F., Li W. (2022). Critical Evaluation of Multifunctional Betaxolol Hydrochloride Nanoformulations for Effective Sustained Intraocular Pressure Reduction. Int. J. Nanomed..

[41] Kim H., Ochoa S.L., Sharma S., Youssif A.A., Thomson B.R., Johnson M., Scott E.A. (2026). Filomicelle-Embedded Composite Hydrogels for Localized Gelation within the Anterior Chamber of the Eye. Small.

[42] Yang H., Zhao M., Xing D., Zhang J., Fang T., Zhang F., Nie Z., Liu Y., Yang L., Li J. (2023). Contact lens as an emerging platform for ophthalmic drug delivery: A systematic review. Asian J. Pharm. Sci..

[43] Fink J.B., Stapleton K.W. (2024). Nebulizers. J. Aerosol Med. Pulm. Drug Deliv..

[44] Kowsigan M., Shetty R., Vyas A. An Enhanced Smart Nebulizer Using IoT in the Treatment of Asthma Patients. Proceedings of the 2023 Innovations in Power and Advanced Computing Technologies (i-PACT).

